# Outcome of recurrent and metastatic small cell carcinoma of the bladder

**DOI:** 10.1186/1471-2490-9-4

**Published:** 2009-06-06

**Authors:** Nabil Ismaili, Pierre Etienne Heudel, Fadi Elkarak, Wafaa Kaikani, Agathe Bajard, Mohammed Ismaili, Hassan Errihani, Jean Pierre Droz, Aude Flechon

**Affiliations:** 1 Department of Medical Oncology, National Institute of Oncology, Rabat-10000, Morocco; 2 Department of Medical Oncology, Centre Léon-Bérard, 28 Rue Laennec, Lyon-69008, France; 3 Department of Biostatistics, Centre Léon-Bérard, 28 Rue Laennec, Lyon-69008, France; 4 Department of Microbiology, Moulay Ismail University, Meknes-50000, Morocco

## Abstract

**Background:**

Bladder small cell carcinoma is an uncommon tumour. Through a retrospective study we will present the evolution of recurrent and metastatic disease and outcome of patients treated at Léon-Bérard Cancer Centre.

**Methods:**

Only 15 patients having recurrent or metastatic bladder small cell carcinoma were treated at Léon-Bérard Cancer Centre between 1996 and 2007. The patients were divided in two groups: a mixed small cell carcinoma group (9 patients) and a pure small cell carcinoma group (6 patients). All the records and informations related to treatment and outcome of the 15 patients were retrospectively analyzed. Various characteristics of small cell carcinoma were investigated.

**Results:**

The median age of the 15 patients having recurrent or metastatic bladder small cell carcinoma and treated at Léon-Bérard Cancer Centre was 63 years and the disease was at stage IV for all cases. Nine patients were treated by chemotherapy. Four patients were treated by local radiotherapy (3 with radiotherapy without previous surgery and 1 with surgery followed by radiotherapy) and chemotherapy. One patient was treated by whole brain radiotherapy. And one patient died before treatment. After 52.4 months median follow up, 12 patients died. Median overall survival was 7.6 months. Survival probability at 1 year was 33%. Median overall survival was 9.9 months in the mixed small cell carcinoma group, and was only 4.6 months in the pure small cell carcinoma group. Survival probability at 1 year in the mixed small cell carcinoma group was 44% as compared to 17% in the pure small cell carcinoma group (Log-rank test: p = 0.228).

**Conclusion:**

Recurrent and metastatic bladder small cell carcinoma is associated with very poor prognosis. The pure bladder small cell carcinoma appears to have poorer outcome than the mixed bladder small cell carcinoma. Chemotherapy using platinum drugs is a mainstay treatment.

## Background

Whereas small cell cancer is a common histological variant accounting for 14% of all primitive cancers arising from the lung [1], small cell carcinoma of the bladder (SCCB) is extremely rare and accounts for less than 1% of all cancers arising from the bladder [2,3]. To our knowledge, 882 cases have been reported in the literature as extra-pulmonary localizations of small cell carcinomas. The first case was described in 1981 by Cramer et al [4]. Small cell carcinoma (SCC) is often, but not always neuroendocrine tumour. The diagnosis of SCCB is based on criteria established by the WHO classification system. Those criteria are identical to those used to diagnose small cell lung cancer (SCLC) [5]. Immunochemical staining can be extremely helpful in establishing the diagnosis. This tumour is associated with a more aggressive behaviour and poorer prognosis than transitional cell bladder carcinoma (TCC), and is mostly diagnosed at advanced stages. Because of the rarity of the disease, no standard treatment had yet been proposed. Treatment algorithms have been extrapolated from the treatment of small cell cancer arising in the lung and often involved chemotherapy regimens using a platinum agent. We conducted a retrospective analysis of all cases of recurrent or metastatic small cell carcinoma of the bladder treated at the Léon-Bérard Cancer Centre over a 12-year period for definition of patient's outcome.

## Methods

All the files of patients with locally advanced or metastatic carcinoma of the bladder that were treated at the Léon-Bérard Cancer Centre between January 1996 and December 2007, were retrospectively reviewed to select all the cases with metastatic or recurrent small cell disease. Patients were considered to have small cell carcinoma of the bladder if pathological examination of their tumour revealed the presence of any small cell component according to the WHO classification [5]. We considered and analysed each patient medical records for further investigations of demographics, clinical stage, histological results, treatments and outcome. Radiological reports were reviewed to determine the stage of the disease caused by recurrent or metastatic small cell carcinoma of the bladder, at the time of diagnosis, using the 2002 TNM classification for genitourinary tumours. Data about the different treatments used: surgery, chemotherapy and radiotherapy were extracted from each patient medical record. The date and site of recurrence and, if applicable, the date and cause of death were also considered. Survival was analyzed statistically in all patients. Overall survival was calculated from the date of diagnosis of recurrent or metastatic disease to the date of death or to the date of last follow up. We retrospectively compared survival between pure small cell carcinoma group (n = 6) and mixed small cell carcinoma group (n = 9), despite of the fact that the two groups of patients were too small and biased by a variety of different therapies. The Kaplan-Meier method was used to calculate median overall survival. The log-rank test was used to evaluate the differences between the two groups. Approval for the study was obtained from Léon-Bérard Cancer Centre, Lyon, France; Date 16/Jul/2007.

## Results

**Figure 1:**
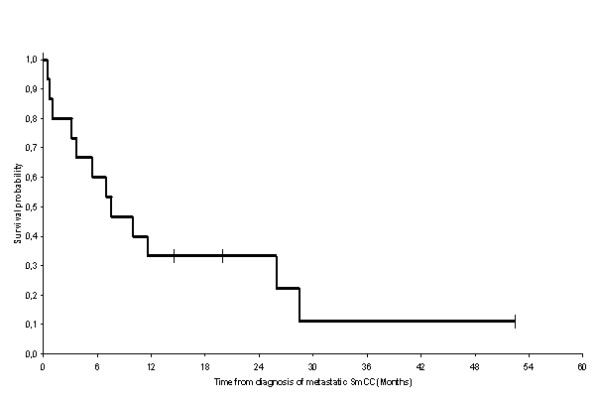
**Overall survival of patients with recurrent or metastatic small cell carcinoma of the bladder (OS): The median duration of overall survival was 7.6 months [n = 15, 12 events (deaths)]**. Survival probability at 1 year was 33%.

**Figure 2:**
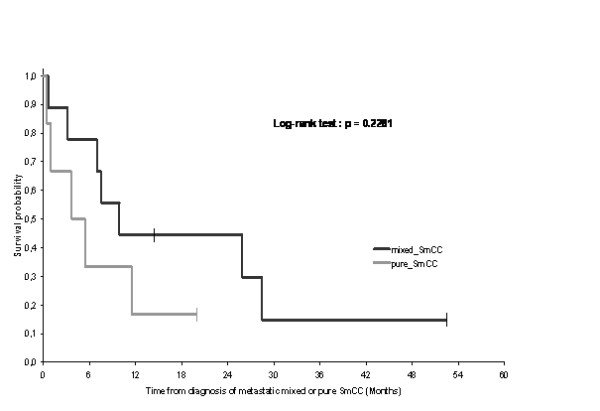
**Overall survival in patients with pure neuroendocrine tumours of bladder vs tumours with mixed histology: The median duration of overall survival was 9.9 months (n = 9, 7 events) in the mixed small cell carcinoma (SmCC) group, as compared with 4.6 months (n = 6, 5 events) in the pure SmCC group**. Survival probability at 1 year in the mixed small cell carcinoma was 44% as compared with 17% in the pure small cell carcinoma group (Log-rank test: P = 0.228).

Between 1996 and 2007, 911 patients with locally advanced or metastatic bladder cancer were treated at the Léon-Bérard Cancer Centre. Only seventeen patients had small cell histology of which 15 had recurrent or metastatic disease. Twelve of the cases analysed in the present study were also included in the study of localized disease and have been the subject of one previous published report [6]. Median age at diagnosis of metastatic or recurrent SCCB was 63 years (range: 45 to 78 years). Fourteen patients were male and one was female. Sixty percent of the patients were smokers. For patients with recurrent disease the pathological diagnosis of SCCB was previously performed before the initial management of the limited stage disease, by cystoscopy and transurethral resection of the bladder tumour (TURBT). For patients with metastatic disease at the time of first presentation, the pathological diagnosis was also performed by cystoscopy and TURBT. Nine patients had both transitional cell and small cell histology (60%), while 6 had exclusively small cell histology (40%). All 15 patients had stage IV disease according to the 2002 TNM classification for genitourinary tumours. Two had limited stage disease and 13 had extensive stage disease in analogy to the two staging system adopted for staging SCLC. Table 1 summarizes the characteristics and clinico-pathological records of patients. Twelve patients were recurrent or metastatic after prior management of the localised stage disease [6]. Eleven of whom were previously treated by radical cystectomy and one by chemotherapy alone because he refused the surgical resection [6]. From theses 12 patients, 2 had local recurrence (one in the neo-bladder and the second in the rectum), 3 had retroperitoneal metastasis, 2 had central nervous system (CNS) metastasis, 2 had liver and retroperitoneal (RP) metastasis, 1 had liver and bone metastasis, 1 had lung and retroperitoneal metastasis and 1 had lung metastasis. Three patients were metastatic at diagnosis: 1 had liver, lung and mediastinum metastasis, 1 had lung and bone metastasis and 1 had bone metastasis. Nine patients were treated by chemotherapy. Three patients were treated by chemotherapy and radiotherapy (sequential treatment). One patient, with rectal recurrence, was treated by surgery, chemotherapy and radiotherapy. One patient, with CNS metastasis, was treated by whole brain radiotherapy. And one patient died before treatment. Tables 2 and 3 summarize the treatments used and the outcome of all patients. Table 4 summarizes chemotherapeutic regimen used to treat patients in the present study. The RECIST criteria were used to determine the response to the treatments mentioned in table 2 and 3. After 52.4-month median follow up, 11 patients died with SCCB and 1 died with sepsis. Median overall survival was 7.6 months. Survival probability at 1 year was 33% (figure 1). Median overall survival was 9.9 months in the mixed small cell carcinoma group, as compared with 4.6 months in the pure small cell carcinoma group. Survival probability at 1 year in the mixed small cell carcinoma was 44% as compared to 17% in the pure small cell carcinoma group (Log-rank test: p = 0.228) (figure 2). Mean survival was 14.7 months for patients (n = 9) treated by chemotherapy and 13.8 months for patients (n = 4) treated by chemotherapy and local radiotherapy treatments (3 with radiotherapy and 1 with surgery followed by radiotherapy).

**Table 1:** Demographic and histopathological characteristics (n = 15).

**Characteristics**	
Age at diagnosis	
Median	63 years
Range	45 – 78 years
Gender	
Male	14
Female	1
Smoking history	
Present	9 (60%)
Absent	6 (40%)
Histologic finding	
SmCC only	6 (40%)
SmCC and TCC	9 (60%)
Stage	
Stage IV(M0)	2
Stage IV(M+)	13

SmCC = small cell carcinoma; TCC = transitional cell carcinoma

**Table 2:** Table showing localisation of recurrence, pathologic and treatment characteristics and outcome of patients with local and metastatic recurrence

**Localisation of recurrence**	**Histology (Mixed or pure SmCC)**	**Treatment**	**Response**	**Survival (months)**	**Death**
Rectum	mixed	Surgery + CT (3EP) + RT	Progressive	7.1	yes
Neo-bladder	Mixed	CT (6MVAC)	PR < 50%	10	yes
RP	Pure	CT (4EP)	Stable	5.5	yes
RP	Mixed	RT (Y 45 Gy) + chemotherapy (4G)	Stable	7.7	yes
RP	Mixed	CT (4GC) + RT (Y 45 Gy)	CR	28.8	yes
CNS	Pure	CT (1 EP + 1 MTX intrathecal)	-	0.4	yes
CNS	Pure	RT (30 Gy)	Progressive	3.7	yes
Liver and RP	Mixed	CT (1GC)	Progressive	0.7	yes
Liver and RP	Mixed	CT (4G)	Progressive	3.1	yes
Liver and Bone	Pure	CT (6EP)	PR > 50%	20.3	no
Lung and RP	Mixed	CT (6EP)	PR > 50%	14.7	no
Lung	Mixed	CT (6EP in first line and 6GC in second line)	PR > 50% (in first line)	26.3	yes

SmCC = small cell carcinoma; CNS = central nervous system; RP = retroperitoneal; EP = etoposide and cisplatin; G = gemcitabine; GC = gemcitabine plus cisplatin; MVAC = methotrexate, vinblastine, doxorubicin and cisplatin; MTX = methotrexate; CT = chemotherapy; RT = radiotherapy; Y = Y field radiotherapy; PR = partial response; CR = complete response, NA = not applicable

**Table 3:** Table showing localisation of metastasis, pathologic and treatment characteristics and outcome of patients with metastatic disease at presentation.

**Localisation of metastasis**	**Histology (Mixed or pure SmCC)**	**Treatment**	**Response**	**Survival (months)**	**Death**
Liver and Lung and Mediastinum	Mixed	CT (22EP)	PR > 70%	51.8	no
Lung and bone	Pure	CT (4EP) + RT (Y 45 Gy) + RT in the bladder (24 Gy).	PR < 50%	11.7	yes
Bone	Pure	none	NA	1	yes

SmCC = small cell carcinoma; CNS = central nervous system; RP = retroperitoneal; EP = etoposide and cisplatin; G = gemcitabine; GC = gemcitabine plus cisplatin; MVAC = methotrexate, vinblastine, doxorubicin and cisplatin; MTX = methotrexate; CT = chemotherapy; RT = radiotherapy; Y = Y field radiotherapy; PR = partial response; NA = not applicable

**Table 4:** Table summarizes chemotherapeutic regimen used to treat patients in the present study.

**Regimen**	**Schedule**	**Drugs and doses**			
EP	On day 1 to 3, repeated after 21 days	Etoposide 120 mg/m^2 ^on day 1 to 3	Cisplatin 80 mg/m^2^, on day 1		
MVAC	On day 1, 2, 15, and 22, repeated after 28 days	Methotrexate 30 mg/m^2 ^on day 1, 15 and 22	Vimblastine 3 mg/m^2 ^on day 2, 15, and 22	Doxorubicin 30 mg/m^2 ^on day 2	Cispatin 70 mg/m^2 ^on day 2
GC	On day 1, 2, 8, and 15, repeated every 28 days	Gemcitabine 1000 mg/m^2 ^on day 1, 8 and 15	Cisplatin 70 mg/m^2^, on day 2		
G	On day 1, 8 and 15 repeated after 21 days	Gemcitabine 1000 mg/m^2^			

EP = etoposide and cisplatin; MVAC = methotrexate, vimblastine, doxorubicin and cisplatin; GC = gemcitabine and cisplatin; G = gemcitabine

## Discussion

Primary SCCB is a rare disease that accounts for less than 1% of all bladder cancers [2,3]. The relatively high incidence of 1.8% reported here might reflect a bias due to the fact that our centre mostly recruits patients with metastatic bladder cancer. Primary SCCB was initially described in 1981 by Cramer et al [4]. Since then, 882 cases of SCCB have been published up to January 2009. Pathogenesis was uncertain; however the multipotent stem cell theory applies best to this case [7-9].

When considering histological tests, SCCB is indistinguishable from its pulmonary counterpart. The diagnosis is accomplished via cystoscopy and histological analysis of the transurethral resection of bladder tumour. The diagnoses were based on the criteria established by the WHO classification system, and were identical to those related to the diagnosis of SCLC [5]. Microscopically, the tumour was composed of sheets of uniformly small, round mitotically active cells with overlapping nuclei and evenly distributed chromatin, lacking prominent nucleoli. Nuclear moulding, tumour necrosis and crush artefact were commonly seen [10]. Immunohistochemical staining and analysis showed that the cells expressed the effect of: 1- the markers of neuroendocrine differentiation, including synaptophysin (expressed at a rate of 66.6 to 76%), neuron-specific enolase (25 to 100%), and chromogranin (22 to 89%); 2- the epithelial markers: EMA (77.7%), Cytokeratine7 (59%), and CAM 5.2. 3 (47 to 66.6%); 3- other markers were less commonly expressed: TTF1 (39 to 50%), C-KIT (22 to 27%) and EGFR (27 to 36%) [3,10-16]. In the present study, we found that most small cell bladder cancers (60%) were mixed with transitional cell carcinoma. In concordance with our results, the mean percentage of mixed SCCB obtained by the analysis of the most important published series was equal to 56% [2,3,6,10,17-22]. Other teams have shown a higher incidence of pure small cell carcinoma [3,18,19,21].

Small cell bladder cancer was highly aggressive as most of the patients (up to 95%) were diagnosed at advanced stage (Stage II or more), from whom 25% were metastatic and two-thirds developed distant recurrence [18,20,23]. Theses finding suggest that the current TNM staging system used for bladder TCC may not be appropriate for SCCB, leading the scientific community to recommend the use of the two staging system (actually in practice): limited and extensive stages in analogy to SCLC [17,21,22,24,25].

Because small cell carcinoma of the bladder was rare, and due to lack of randomized controlled trials, no standard treatment of this disease was proposed. However, chemotherapy played a prominent role in the management of these tumours. The prognostic of SCCB was poor even in limited stage disease [20]. According to 2 large series, five years survival in all stages ranged between 16 and 25% [18,20]. In our institution we conducted a retrospective study of 14 localised small cell carcinoma of the urinary bladder. The disease free survival was equal to 5.7 months and the overall survival was equal to 29.5 months. Two years survival was equal to 56% [6]. The prognostic of patients with stage IV disease is very poor. In the Mayo Clinic study, 19 patients were diagnosed with stage IV disease (with or without distant metastasis), only 2 were survivors. The median overall survival for patients with stage IV disease was 11 months and the one year survival rate for theses patients was 36.8%. The results obtained in our series were in concordance with previous Mayo Clinic results and showed a 7.6-month median survival and 33% survival probability at 1 year [18].

Treatment algorithms have been extrapolated from the treatment of SCLC and often involved chemotherapy. The gold standard chemotherapy for patients with good performance status SCLC was platinum based regimen, typically cisplatin-etoposide [26-28]. In analogy to SCLC, cisplatin-etoposide regimen was mostly used in the management of SCCB either in LS or ES [17,18,23]. In ES SCLC, irinotecan-cisplatine regimen was shown to be an effective treatment [29,30]. Other chemotherapy regimens including etoposide-cisplatine alternating protocol either with ifosfamide-doxorubicin or with cyclophosphamide, doxorubicin and vincristine (CAV), as well as single agents, including paclitaxel, irinotecan, topotecan, and doxorubicin, have all been used in SCCB [18,23]. The MD Anderson group showed that preoperative chemotherapy with a neuroendocrine regimen was more likely to successfully eradicate the small cell component compared to regimens typically used for transitional cell carcinoma. In fact, within the 12 patients treated with a neuroendocrine regimen only 2 had small cell carcinoma present at cystectomy. However, for those 9 patients treated with a transitional cell carcinoma regimen (methotrexate, vinmblastine, doxorubicin, and cisplatin: MVAC regimen) 6 had small cell carcinoma still present at cystectomy [23]. Consequently, this group recommend the protocols used in the neuroendocrine tumours containing etoposide and cisplatin or ifosfamide and doxorubicin for both histological types: pure small cell carcinoma and mixed small cell carcinoma of the bladder. Other authors recommended a regimen covering both small cell component and transitional cell component for mixed SCCB: the addition of taxane or ifosfamide to the standard platinum plus etoposide regimen may be considered [24]. Table 5 summarizes the most used regimen in the management of SCCB in analogy to SCLC. Similarly, four patients in our series were successfully managed by chemotherapy using etoposide plus cisplatin regimen with 28.3 months mean survival. In addition, the patients treated with local treatment (surgery and/or radiotherapy) plus chemotherapy had the same mean survival as patients treated with chemotherapy alone (13.8 vs 14.7 months). These results suggested that chemotherapy was more significant than local treatments. In analogy to SCLC, radiotherapy can be used to palliate brain metastases, symptomatic bone metastases and cord compression [31]. One of our patients with brain metastasis only, was managed with palliative whole brain radiotherapy only, but the disease progressed and the patient was dead 3.7 months later.

**Table 5:** Table summarizes chemotherapeutic regimen used in the management of SCCB

**Regimen**	**Schedule**	**Drugs and doses**				
**First line chemotherapy: Mixed and pure SCC**						

EP [18,21,22]	On day 1 to 3, repeated after 21 days	Etoposide 120 mg/m^2 ^on day 1 to 3	Cisplatin 80–100 mg/m^2^, on day 1			
IP [28,29]	On day 1, 8, and 15, repeated every 28 days	Irinotecan 60 mg/m^2 ^on days 1, 8 and 15	Cisplatin 60 mg/m^2 ^on day 1			
VIP [21]	On day 1 to 4, repeated after 21 days	Ifosfamide 1.2 g/m^2^, on day 1 to 4	Etoposide 75 mg/m^2 ^on day 1 to 4	Cisplatin 20 mg/m^2 ^on day 1 to 4		
EP/CAV [22]	Alternative regimen: PE on day 1 to 3 repeated after 42 days and CAV on day 1 repeated after 42 days	Etoposide 100 mg/m^2 ^on day 1 to 3	Cisplatin 80 mg/m^2^, on day 1	Cyclophosphamide 800 mg/m^2^	Doxorubicin 50 mg/m^2^	Vincristine 1.4 mg/m^2^
						
**First line: mixed SCC**						

MVAC [18]	On day 1, 2, 15, and 22, repeated after 28 days	Methotrexate 30 mg/m^2 ^on day 1, 15 and 22	Vimblastine 3 mg/m^2 ^on day 2, 15, and 22	Doxorubicin 30 mg/m^2 ^on day 2	Cispatin 70 mg/m^2 ^on day 2	
						
**Second line chemotherapy in analogy to SCLC**						

IV Topotecan [18]	On day 1 to 5, repeated every 21 days	Topotecan 1.5 mg/m^2 ^on day 1 to 5				
CAV	On day 1, repeated every 21 days	Cyclophosphamide 800 mg/m^2^	Doxorubicin 50 mg/m^2^	Vincristine 1.4 mg/m^2^		
TP	On day 1 to 5, repeated every 21 days	Topotecan 0.75 mg/m^2 ^on day 1 to 5	Cisplatin 60 mg/m^2 ^on day 1			

EP = etoposide and cisplatin; VIP = etoposide, ifosfamide and cisplatin: PE/CAV = cisplatin and etoposide/cyclophosphamide, doxorubicin and vincristine; CEA = cyclophosphamide, etoposide and doxorubicin; CaE = carboplatin and etoposide; MVAC = methotrexate, vinmblastine, doxorubicin and cisplatin; IV = intravenous; SCC = small cell carcinoma; SCLC = small cell lung cancer

Considering the generally poor prognosis of SCCB, novel therapeutic strategies are needed to improve outcomes of patients. Targeted therapies are now established for several diseases, but have not yet been investigated in SCCB. C-KIT protein expression has been reported in 27% of cases of SCCB suggesting the possibility to consider the therapeutic use of STI-571, a small molecule inhibitor of C-KIT kinase activity, in patients with c-kit positive tumours [15]. However, STI-571 has been tried in treatment of SCLC and found to be ineffective [32-34].

Finally, we found that pure small cell carcinoma tended to have poorer outcome than mixed small cell carcinoma of the bladder. The median duration of survival was 9.9 months in the mixed small cell carcinoma group, as compared with 4.6 months in the pure small cell carcinoma group, but the difference was not statistically significant (Log rank test: p = 0.228) (Figure 2). In two series, mixed histology tended to do better than pure neuroendocrine tumour [6,19].

## Conclusion

Small cell carcinoma of the bladder is an uncommon tumour. Recurrent and metastatic disease was associated with very poor prognosis. The pure small cell carcinoma appeared to have poorer outcome than the mixed small cell carcinoma of the bladder. In the absence of prospective studies, the best treatment of this disease cannot be established with certainty. From our study and from the literature, we conclude that platinum-based chemotherapy is the mainstay treatment for recurrent and metastatic disease.

## Competing interests

The authors declare that they have no competing interests.

## Authors' contributions

All authors read and approved the final manuscript.

NI: conception and design, acquisition of data, analysis and interpretation of data, statistical analysis, literature review, drafting the manuscript and revising it critically for important intellectual content; PEH: acquisition and analysis of data; FE: acquisition and analysis of data; WK: acquisition of data; AB: statistical analysis; MI: review of finale manuscript; HE: review of finale manuscript; JPD: review of finale manuscript; AF: review of final manuscript.

## Pre-publication history

The pre-publication history for this paper can be accessed here:


